# Data supporting the investigation of interaction of biologically relevant proteins with ZnO nanomaterials: A confounding factor for in vitro toxicity endpoints

**DOI:** 10.1016/j.dib.2019.103795

**Published:** 2019-03-01

**Authors:** Emilie Da Silva, Yahia Kembouche, Ulla Tegner, Anders Baun, Keld A. Jensen

**Affiliations:** a The National Research Center for the Working Environment, Lersø Parkallé 105, Copenhagen, Denmark; b Department of Environmental Engineering, Technical University of Denmark, Bygningstorvet 115, Kgs. Lyngby, Denmark

**Keywords:** BSA, bovine serum albumin, DLS, dynamic light scattering, DMEM, Dulbecco's modified eagle's medium, FBS, fetal bovine serum, ILs, interleukins, LDH, lactate dehydrogenase, MN, manufactured nanomaterials, OD, optical density

## Abstract

Test materials, like manufactured nanomaterials (MN), may interact with serum proteins, interleukins (IL) and lactate dehydrogenase (LDH) and cause measurement artefacts as a result of e.g., physical adsorption and electrostatic forces, and/or interaction with dissolved species or conditional chemical changes during testing. In this article, data are given on the zeta-potentials of two manufactured ZnO nanomaterials (NM-110 and NM-111) dispersed in 0.05% w/v Bovine Serum Albumin (BSA) water batch dispersions and in Ham's F12 nutrient mixture added Fetal Bovine Serum (FBS), penicillin, and streptomycin and particle free mediums (cHam's F12). Data on the Zeta-potential and the iso-electrical point of lactate hydrogenase in pure Ham's F12 nutrient mixture is also provided. The percentage of added IL-6, IL-8 and LDH remaining after 24-h incubation in cHam's F12 are given as function of MN concentrations. Finally data from thermodynamic chemical reaction modeling of changes in pH and Zn-speciation during dissolution of ZnO or dissolved ZnCl_2_ additions to Ham's F12 using Geochemist Workbench^®^ are given. For further information, data interpretation and discussion please refer to the research article “Interaction of biologically relevant proteins with ZnO nanomaterials: a confounding factor for in vitro toxicity endpoints” (E. Da Silva et al. 2019).

**Table:** Specifications table

Subject area	*Chemistry, Biology*
More specific subject area	*Nanotoxicology in vitro*
Type of data	*Table, graph*
How data was acquired	*Zeta potential was measured using dynamic light scattering analysis (DLS, Zetasize Nano ZS, Malvern Instruments Ltd., Malvern, United Kingdom). Data recording and treatments were made using the Zetasizer software v. 7.11 and 7.12 (Malvern Instruments Ltd., Malvern, United Kingdom).* *The quantification of LDH absorbance was performed using LDH Cytotoxicity Detection Kit's instructions (Roche Diagnostics GmBH, Roche Applied Science, Mannheim, Germany). The amount of IL-6 and IL-8 was measured by ELISA (BD Pharmingen kit, Cat. No. 555220 and 555244 respectively; BD Biosciences, Lyngby, Denmark).* *Change in pH and ionic species during addition of either ZnO or dissolved ZnCl*_*2*_*to Ham's F12 was assessed using the React program and plotted using the Gtplot Apps in Geochemist Workbench® v. 11.0.*
Data format	*Raw data for zeta-potential measurements are depicted in the graphs.* *LDH activity and protein levels are expressed as the percentage of their reference values in the test medium without ZnO Manufactured Nanamaterials (MN).* *Calculated pH and ionic species concentrations are depicted in graphs as functions of added ZnO, ZnCl*_*2*_*and dissolved Zn*^*2+*^
Experimental factors	*LDH activity and protein levels are expressed as the percentage of their reference values in the test medium without ZnO MN.*
Experimental features	*Zeta potential of NM-110 and NM-111 was measured in the batch dispersion and at different MN concentrations in the test medium.* *LDH surface charge and isoelectric point were determined by zeta potential measurements as function of pH.* *Changes in pH and Zn-species during dissolution and increased dissolved Zn concentrations were estimated by thermodynamic chemical modeling.*
Data source location	*Copenhagen, Denmark, The National Research Centre for the Working Environment*
Data accessibility	*Data are directly available from tables and graphs in this data in brief article*
Related research article	Emilie Da Silva, Yahia Kembouche, Ulla Tegner, Anders Baun, Keld A. Jensen. Interaction of biologically relevant proteins with ZnO nanomaterials: a confounding factor for in vitro toxicity endpoints. Toxicology in Vitro, Volume 56, April 2019, Pages 41-51 [1]*.*

**Table:** 

**Value of the data** • The surface charge of MN and the adsorption of serum proteins from the dispersion medium on to ZnO MN are portrayed. This is important because these parameters can affect MN fate and interaction with specific protein assay^1−6^ • It is important to account for interaction between the MN and measured biomolecules, e.g. interleukins, LDH, lipids, and reactive oxygen species, which could lead to artefacts in the identification and quantification of biological response • The value of thermodynamic chemical reaction modeling is demonstrated and show that an increase in dissolved Zn from ZnO MN in cHAM's F12 can lead to an increase in pH, reducing the negative surface charge towards neutral • Parameters that may be determinants for LDH interaction (Zn-ion concentration due to dissolution of ZnO MN in the test medium, changes in pH, and changes in protein zeta potentials) can be shown to vary during incubation

## 1 Data

Table 1 lists the zeta-potentials of NM-110 and NM-111 (uncoated and coated ZnO manufactured nanomaterials, respectively [1], [2]) in cHam's F12 test medium over time (0, 30 and 60 minutes) at 10, 320 and 640 μg ZnO/mL and the 0.05% w/v BSA water batch dispersions with 2560 μg ZnO/mL. The zeta potentials of the particle free mediums are shown for reference. The pH values in the dispersions were close to neutral (NM-110: pH = 7.0 ± 0.3; NM-111: pH = 6.8 ± 0.3) during measurements.

**Table 1:** Zeta potential of NM-110 and NM-111 in cHAMs F12 as function of MN concentration and time as well as in the cHAMs F12 and the 0.05% BSA-water batch dispersion with and without NM-110 and NM-111.

Test material	MN concentration (μg/mL)	Time (min)	Zeta potential (mV, mean ± sd)
NM-110 in cHAMs F12	10	0	0.08 ± 1.18
	10	30	0.06 ± 1.72
	10	60	−0.41 ± 0.90
	320	0	−0.47 ± 0.87
	320	30	−0.79 ± 0.65
	320	60	−0.10 ± 1.30
	640	0	0.31 ± 0.93
	640	30	0.99 ± 1.01
	640	60	0.22 ± 1.03
NM-111 in cHAMs F12	10	0	−11.20 ± 1.03
	10	30	−12.82 ± 0.47
	10	60	−13.10 ± 0.80
	320	0	−12.92 ± 0.74
	320	30	−13.62 ± 0.35
	320	60	−14.15 ± 0.93
	640	0	−13.13 ± 0.71
	640	30	−13.77 ± 0.98
	640	60	−14.32 ± 1.04
cHam's F12	–	0	−10.44 ± 2.00
	–	30	−12.43 ± 1.01
	–	60	−12.00 ± 1.10
NM-110 in 0.05% BSA	2560	0	−0.02 ± 0.39
	2560	30	−0.17 ± 0.32
	2560	60	−0.16 ± 0.34
NM-111 in 0.05% BSA	2560	0	0.07 ± 0.38
	2560	30	−0.04 ± 0.29
	2560	60	0.01 ± 0.27
	–	0	−20.70 ± 0.14
0.05% BSA	–	30	−23.25 ± 1.77
	–	60	−24.40 ± 1.13

Table 2 lists the percentage of the initially added levels of IL-6 (500 pg/mL), IL-8 (4000 pg/mL) and LDH (100 ng/mL) after incubation with NM-110 and NM-111 at eight different doses (0–640 μg/mL) in cHam's F12: The LDH data (OD_LDH_) are given as the optical density for LDH expressed as a percentage of optical density of the medium reference OD_ref_ without MN. IL-6 and IL-8 concentrations are given as the percent of the initially dosed concentrations.

**Table 2:** LDH, IL-6 and IL-8 levels after incubation with NM-110 and NM-111 for 24 h at 37 °C.

ZnO MN	MN concentration (μg/mL)	mean ± sd		
		LDH (% OD_ref_)	IL-6 (% initial concentration)	IL-8 (% initial concentration)
NM-110	0	100 ± 0.8	100 ± 2.4	100 ± 1.8
	10	40.5 ± 6.9^a^	100.2 ± 2.8	96.8 ± 2.2
	20	34.2 ± 5.6^a^	98.9 ± 3.7	97.9 ± 2.8
	40	31.5 ± 3.9^a^	100.2 ± 6.3	97.0 ± 4.2
	80	30.1 ± 3.4^a^	100.6 ± 5.1	98.6 ± 6.8
	160	28.3 ± 2.7^a^	97.8 ± 5.2	95.4 ± 4.0
	320	27.6 ± 2.6^a^	98.5 ± 4.0	96.7 ± 3.6
	640	26.5 ± 1.5^a^	96.8 ± 4.4	95.4 ± 6.8
NM-111	0	100 ± 0.5	100 ± 2.1	100 ± 2.1
	10	47.2 ± 9.7^a^	98.9 ± 4.3	96.8 ± 5.5
	20	35.9 ± 4.3^a^	99.2 ± 9.1	95.8 ± 7.4
	40	32.3 ± 3.0^a^	99.1 ± 6.5	100.6 ± 9.8
	80	30.3 ± 3.4^a^	100.3 ± 6.0	99.2 ± 12.0
	160	28.0 ± 3.3^a^	96.1 ± 9.3	98.9 ± 13.4
	320	26.4 ± 3.0^a^	96.5 ± 8.2	101.7 ± 9.8
	640	25.4 ± 3.4^a^	97.2 ± 6.6	107.7 ± 15.9

sd = standard deviation, obtained from three independent experiments with two replicates each.

a Denotes a statistically significant difference with the baseline (ANOVA, p-value <0.001).

**Fig. 1:**
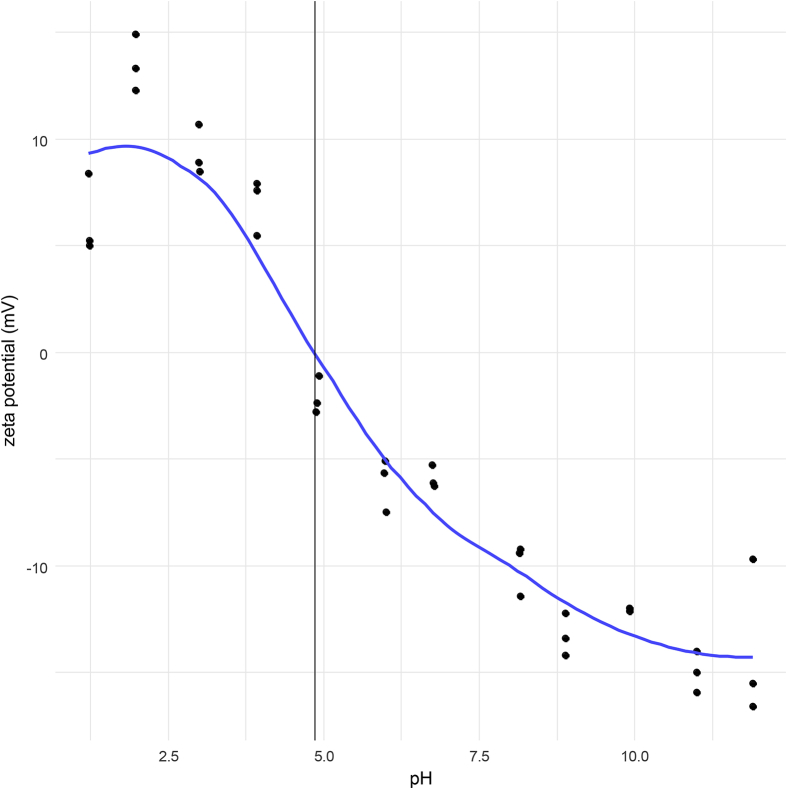
Zeta potential of LDH (10000 ng/mL) in cHam's F12 versus pH. The vertical line represents the isoelectric point where the surface charge is neutral.

Fig. 1 shows the zeta-potential data as function of pH and the iso-electrical point of LDH at pH = 4.67 (vertical line, triplicates) in pure Ham's F12 nutrient mixture. A concentration of 10000 ng LDH/mL was used to allow good zeta-potential measurements. 1 M HCl and 1 M NaOH were used for pH-titrations.

**Fig. 2:**
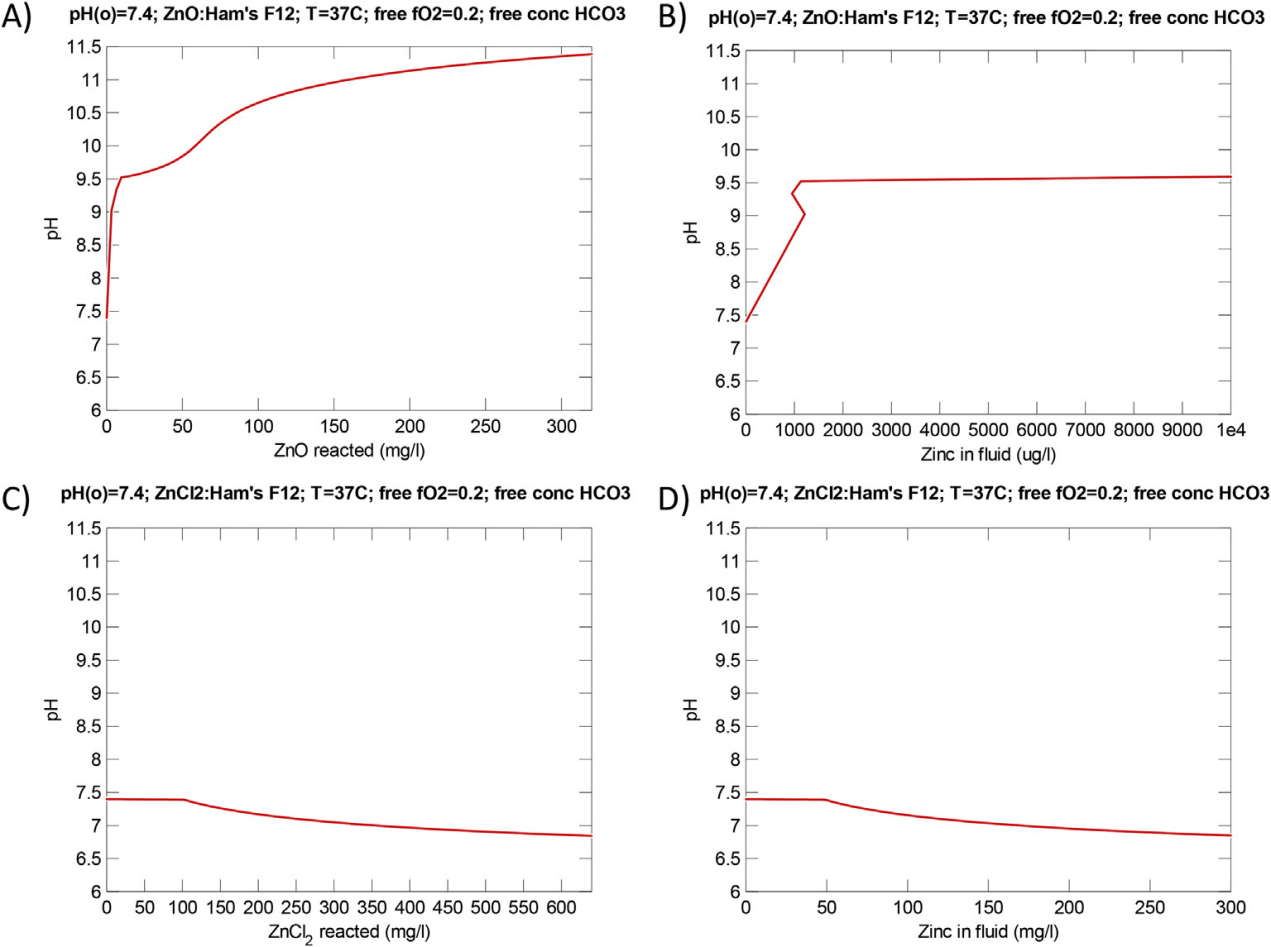
Change in pH in Ham's F12 nutrient mixture at 37 °C and CO_2_ balanced atmosphere using an initial pH 7.4 and an oxygen fugacity of 0.2 at different conditions. A. pH-change during simulated reaction with up to 320 μg/mL ZnO. B. pH-change as a function of measured Zn concentration due to dissolution of ZnO. C. pH-change during simulated reaction with up to 640 μg/mL ZnCl_2_. D. pH-change as a function of the resulting Zn concentration (<307 μg/mL) due to dosing of up to 640 μg/mL ZnCl_2_. For all plots, calculations were made using the React and Gtplot Apps in Geochemist's Workbench^®^ version 11 [3].

Fig. 2 shows the pH-variation as function of the amount of ZnO (Fig. 2A and B) and ZnCl_2_ (Fig. 2C and D) reacted and as function of Zn-concentrations in Ham's F12 nutrient mixture calculated using the React program in the Geochemist Workbench^®^ [3]. The pH may increase to approximately 9.5 during dissolution of only 10 μg ZnO/mL (Fig. 2A and B). Opposite, a slight reduction in pH from pH 7.4 to pH 6.7 occurs with increased amounts of dissolved ZnCl_2_ (Fig. 2C and D).

**Fig. 3:**
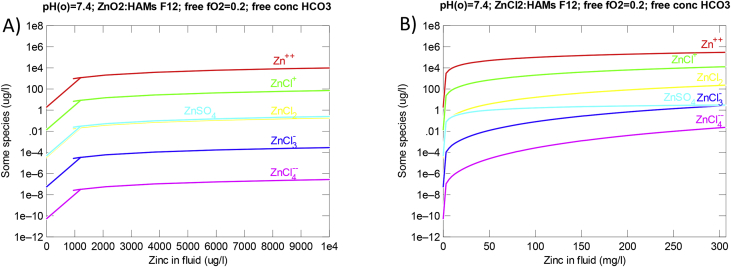
Change in Zn-species concentrations as function of dissolved Zn-concentrations in Ham's F12 nutrient mixture at 37 °C and CO_2_ balanced atmosphere using an initial pH 7.4 and an oxygen fugacity of 0.2. A. Zn-species concentrations during simulated dissolution of up to 320 μg/mL ZnO. B. Zn-species concentrations during simulated dissolution with up to 640 μg/mL ZnCl_2_. D. For all plots, calculations were made using the React and Gtplot Apps in Geochemist's Workbench^®^ version 11 [3].

Fig. 3 shows the data from calculations of the Zn speciation in Ham's F12 nutrient mixture during dissolution of ZnO and ZnCl_2_. More than 99.3% (by weight) of the dissolved Zn will occur as Zn^2+^ during dissolution of ZnO (Fig. 3A). Slightly less (>95.8% by weight) will be Zn^2+^ during dissolution of ZnCl_2_ (Fig. 3B).

## 2 Experimental design, materials and methods

Two ZnO MN test materials; NM-110 (uncoated ZnO) and NM-111 (tri-ethoxy-silane-coated ZnO) and ZnCl_2_ originating from the from the Sponsorship Programme for the Testing of Manufactured Nanomaterials organized by the OECD Working Party on Manufactured Nanomaterials [4] were used here. The main research article [1] is referred to for compiled data on the ZnO MN. ZnCl_2_ was purchased from Merck (EMSURE^®^ ACS, Reag. Ph Eur, catalogue no. 108816).

Batch dispersions of the test materials were produced by dispersing 2.56 mg/mL in sterile filtered 0.05% (v/v) BSA water (Nanopure Diamond UV) by probe-sonication as described in the NANOGENOTOX batch dispersion protocol [5]. Sonication was performed using a Branson Sonifier S-450D (Branson Ultrasonics Corp., Danbury, CT, USA) operated for 16 min at 400 W and 10% amplitude using a 13 mm disruptor horn. For further details please see Jensen et al. [5].

The complete test medium (cHam's F12) consisted of Ham's F12 nutrient mixture (ThermoFisher Scientific, Hvidovre, Denmark) with 1% v/v penicillin/streptomycin (10000 U/mL and 10 mg/mL respectively; In Vitro A/S, Fredensborg, Denmark) and 10% v/v of inactivated Fetal Bovine Serum (USA grade; In Vitro A/S, Fredensborg, Denmark). For interaction studies cHam's F12 was added 500 pg/mL IL-6 standard (National Institute for Biological Standards and Controls, Hertforshire, UK), 4000 pg IL-8 standard (National Institute for Biological Standards and Controls, Hertforshire, UK; final concentration), and 100 ng/mL rabbit muscle LDH (Sigma-Aldrich, Brøndby, Denmark). Please see Da Silva et al. [1] for further details.

Zeta potential measurements were completed using a Zetasizer Nano ZS (Malvern Instruments Ltd., Malvern, United Kingdom) controlled by Zetasizer software v. 7.11 and 7.12. For determination of the iso-electrical point, the pH was adjusted using 1 M HCl, (grade AVS TITRINORM Reagent Ph.Eur. chapter 4.2.2, USP, NF, VWR International S.A.S., 42250 Briane, France) and 1M NaOH, (grade AVS TITRINORM Reagent Ph.Eur. chapter 4.2.2, USP, NF, VWR International S.A.S., 42250 Briane, France) dosed using the MPT-2 Titrator (Malvern Instruments Ltd., Malvern, United Kingdom). Please see Da Silva et al. [1] for further details.

IL-6, IL-8 and LDH levels in cHam's F12 with and without test materials were determined after 24 h incubation in darkness at 37 °C and a 5% CO_2_ atmosphere with a 95% relative humidity in a cell incubator (CelCulture^®^ CO_2_ Incubator, ESCO Medical, Egaa, Denmark) maintaining a temperature of 37 °C and a 5% CO_2_ atmosphere with a 95% relative humidity in the dark without shaking. Suspensions were collected, centrifuged and prepared for spectrophotometric analysis of interleukine (BD Pharmingen kit, Cat. No. 555220 and 555244 respectively; BD Biosciences, Lyngby, Denmark) and LDH (Roche Diagnostics GmBH, Roche Applied Science, Mannheim, Germany) using an ELISA reader as described in Da Silva et al. [1].

Thermodynamic chemical reaction modeling of dissolved Zn concentration associated effect on pH and Zn-ion speciation after addition of ZnO and ZnCl_2_ was completed using the Geochemist Workbench^®^ v. 11.0 [3]. Please see Da Silva et al. [1] for further details on medium composition and conditions defined for generating the data.

Statistical analysis, tables and graphs were made using R (version 3.4.3) and Gt-plot in Geochemist Workbench^®^ v. 11.0. Statistical significance was set at p-value < 0.05. Each treatment was repeated in duplicate and each experiment replicated three times.
